# Supplementary material for: Yiai Fuzheng decoction inhibits triple-negative breast cancer by remodeling the immune microenvironment
Source: Front Immunol. 2025 Sep 30;16:1615631. doi: 10.3389/fimmu.2025.1615631 (PMC12518410; doi:10.3389/fimmu.2025.1615631)
Supplement: Supplementary file 2 [file DataSheet1.zip › Supplementary File 2/Treatment/C_vs_H/07_Pathway_Analysis/Pathway_Analysis/Sig_Pathways_Name/Lysine degradation.pdf]

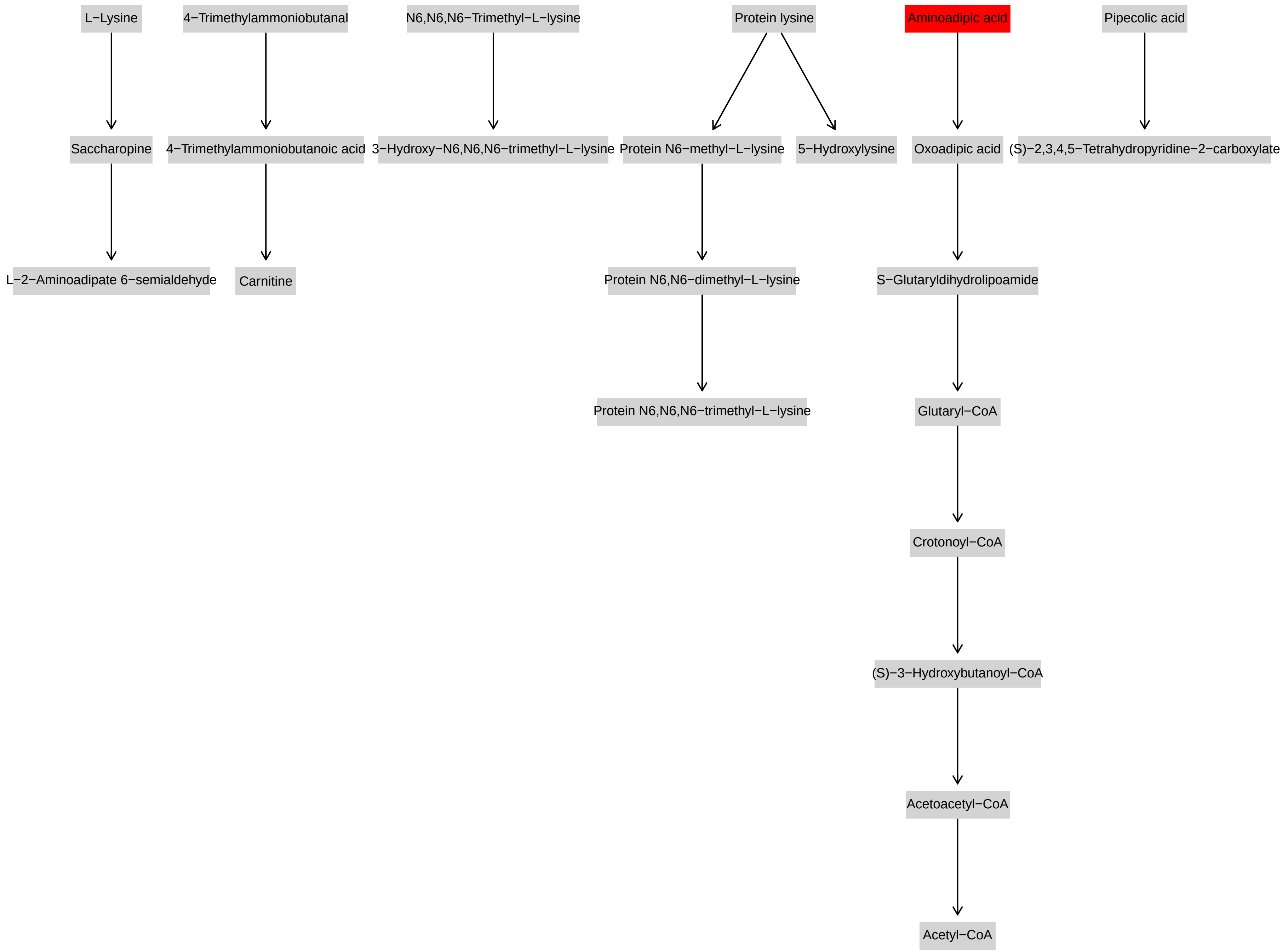
L−Lysine
4−Trimethylammoniobutanal
N6,N6,N6−Trimethyl−L−lysine
Protein lysine
Aminoadipic acid
Pipecolic acid
Saccharopine
4−Trimethylammoniobutanoic acid
3−Hydroxy−N6,N6,N6−trimethyl−L−lysine
Protein N6−methyl−L−lysine
5−Hydroxylysine
Oxoadipic acid
(S)−2,3,4,5−Tetrahydropyridine−2−carboxylate
L−2−Aminoadipate 6−semialdehyde
Carnitine
Protein N6,N6−dimethyl−L−lysine
S−Glutaryldihydrolipoamide
Protein N6,N6,N6−trimethyl−L−lysine
Glutaryl−CoA
Crotonoyl−CoA
(S)−3−Hydroxybutanoyl−CoA
Acetoacetyl−CoA
Acetyl−CoA
